# A multidisciplinary, AI‐supported quality improvement intervention to manage polypharmacy in aging people with HIV

**DOI:** 10.1111/hiv.70234

**Published:** 2026-04-11

**Authors:** Jovana Milic, Antonia Pugliese, Michela Belli, Gian Luca Lonardi, Caterina Ruffilli, Tommaso Albano, Marco Visicaro, Martina Ricciardetto, Pierluigi De Cosmo, Chiara Mussi, Francesca Gandolfi, Cristina Mussini, Costantino Grana, Giovanni Guaraldi

**Affiliations:** ^1^ Department of Surgical, Medical, Dental and Morphological Sciences University of Modena and Reggio Emilia Modena Italy; ^2^ Distribuzione Diretta AUSL Modena, Dipartimento Farmaceutico Interaziendale Modena Italy; ^3^ Engineering Informatics Modena Italy; ^4^ School of Medicine University of Modena and Reggio Emilia Modena Italy; ^5^ Infectious Diseases Clinic Azienda Ospedaliero‐Universitaria, Policlinico of Modena Modena Italy; ^6^ Infologic Srl Padova Italy; ^7^ Department of Biomedical, Metabolic and Neural Sciences and Center for Gerontological Evaluation and Research University of Modena and Reggio Emilia Modena Italy; ^8^ Enzo Ferrari Department of Engineering University of Modena and Reggio Emilia Modena Italy

**Keywords:** aging, inappropriate prescription, older people with HIV, polypharmacy, quality improvement

## Abstract

**Objectives:**

Aging people with HIV are increasingly affected by multimorbidity and polypharmacy, which heighten the risk of drug–drug interactions (DDIs) and potentially inappropriate medications (PIMs). This study evaluated a multidisciplinary, AI‐supported quality improvement intervention designed to optimize polypharmacy management in older people with HIV.

**Methods:**

People with HIV aged ≥50 years attending the Modena HIV Metabolic Clinic (MHMC) were invited to submit photos of their medications via WhatsApp. Images were processed by AI for optical character recognition and automatically reconciled with the electronic patient chart (EPC). AI recognition accuracy was 94% when validated against manual review. Pharmacists reviewed AI‐generated reports from the NavFarma® decision support system, generated alerts for PIM, defined according to Beers and the STOPP/START criteria, DDIs, anticholinergic burden (ACB), and risks of QTc prolongation and nephrotoxicity. Primary outcome was agreement between patient‐reported and EPC‐recorded medications. Secondary outcomes included pill burden, total prescribed drugs and actionable alerts.

**Results:**

Of 181 participants (median age 63 years; 72% male), 111 (61.3%) showed complete agreement between EPC and patient lists, while 70 (38.7%) had discrepancies. Pharmacist evaluation identified major DDIs in 70.4% of cases, ACB in 26.5%, QTc‐prolonging drugs in 81.6% and nephrotoxic agents in 95.9%. Participants with ≥10 total prescribed drugs had higher frailty, pill burden and PIM.

**Conclusions:**

AI‐assisted medication reconciliation combined with pharmacist review improved the identification of PIM and medication‐related risks, supporting safer prescribing in people with HIV. This model aligns with international calls to improve prescribing safety and offers a scalable framework for integrating digital tools into multidisciplinary HIV care.

## BACKGROUND

As people with HIV age, the clinical management of their health becomes increasingly complex due to the rising prevalence of multimorbidity and the resulting burden of polypharmacy [1, 2]. With the success of antiretroviral therapy (ART), HIV has become a chronic condition, leading to a growing population of older people with HIV who often face a constellation of age‐related comorbidities, including cardiovascular disease, diabetes, osteoporosis and cognitive impairment [3, 4]. Managing these comorbidities typically requires the use of multiple concomitant medications, which significantly increases the risk of adverse drug reactions (ADRs), drug–drug interactions (DDIs), increased pill burden, reduced adherence and overall poorer health outcomes [5].

Polypharmacy, commonly defined in the context of HIV as the concurrent use of five or more medications excluding ART, may be appropriate when multimorbidity is present. However, inappropriate polypharmacy, defined as the use of medications that are no longer clinically indicated or prescribed at incorrect doses, remains a major challenge. In this study, potentially inappropriate prescriptions were defined according to explicit criteria embedded in the NavFarma® clinical decision support system, primarily based on the 2023 American Geriatrics Society Beers Criteria and the STOPP criteria. These criteria were applied to the reconciled medication list, taking into account age, comorbidities, renal function and concomitant drug exposure, to identify medications with an unfavourable risk–benefit profile in older people with HIV [6]. Appropriate polypharmacy entails the intentional use of multiple medications to achieve defined therapeutic goals, tailored to the patient's clinical condition, preferences and life expectancy [7]. In contrast, inappropriate polypharmacy involves medications without a clear indication, those that may no longer provide benefit, or combinations that increase the risk of ADRs, therapeutic duplication, reduced adherence and mortality [8].

Several interventions have been proposed to mitigate these risks, including pharmacist‐led medication reviews, deprescribing protocols supported by decision algorithms and the use of technology to facilitate medication reconciliation [9]. Evidence shows that such interventions can improve prescribing accuracy, reduce adverse outcomes and enhance patient satisfaction, especially when embedded within multidisciplinary care models [10].

Despite growing awareness, important gaps remain in the management of polypharmacy among people with HIV. These include insufficient integration of medication review into HIV care workflows, limited training in geriatric pharmacotherapy for HIV specialists and the underuse of decision support tools tailored to this population. Furthermore, patient engagement in medication‐related decisions is often suboptimal, limiting the effectiveness of deprescribing efforts.

Quality improvement (QI) strategies aimed at rationalizing medication use are thus essential components of comprehensive HIV care in aging populations. Yet, integrating these practices into routine clinical workflows remains challenging, especially in resource‐constrained or time‐pressured settings.

The aim of this study was to describe the implementation of an innovative, multidisciplinary, AI‐supported QI intervention at the Modena HIV Metabolic Clinic (MHMC), designed to optimize medication management in aging people with HIV. Specifically, the study addressed: (i) drug recognition and reconciliation, assessing the agreement between medications recorded in the electronic patient chart (EPC) and those reported by the patient; (ii) quantification of total number of active pharmacologic agents and pill burden, as well as identification of actionable alerts generated per patient, including DDIs, inappropriate prescriptions, anticholinergic burden (ACB) and exposure to drugs associated with QTc prolongation and nephrotoxicity; and (iii) the rate of physician acceptance of pharmacist recommendations and their integration into patient care plans. The overarching goal was to enhance medication safety, improve treatment efficacy and promote person‐centred care through the integration of digital tools, pharmacist‐led recommendations and personalized clinical decision‐making.

## METHODS

### Study design and setting

This was a cross‐sectional observational study including ART‐experienced people with HIV ≥50 years attending the MHMC, a tertiary‐level, multidisciplinary specialty care clinic embedded within the Infectious Diseases Unit, providing comprehensive geriatric, metabolic and neurocognitive evaluations for aging people with HIV. The MHMC does not deliver primary care but focuses on the management of complex multimorbidity, polypharmacy and frailty through an integrated specialist model. Potential participants were identified through the clinic's electronic scheduling system and contacted approximately 1 week before their scheduled visit via an automated WhatsApp message inviting them to submit photos of their current medications. During the study period, 297 eligible people with HIV were invited, and 181 (60.9%) completed the medication submission process and were included in the analysis. The study protocol was approved by the Area Vasta Emilia Nord Ethics Committee (protocol number 0028673/23), and all participants provided written informed consent.

### QI project

This QI project consisted of (i) real‐time medication data collection through AI‐enhanced recognition and subsequent generation of reconciliation list, (ii) AI integration of collected data with NavFarma database, (iii) pharmacist review and recommendations and (iv) multidisciplinary decision‐making. A detailed description of the project is detailed in the Annex 1.

Patients submitted photos of their medications via WhatsApp 1 week before their scheduled visit. Images were processed through an AI‐based system without human intervention; human review occurred only at subsequent stages, including physician validation and pharmacist evaluation of AI‐generated alerts (accuracy: 94%) that coded medications and integrated them into the EPC. Details of the process are described in the Annex 2 (AI‐Supported Medication Collection Tool). The standardized medication dataset was then integrated with the NavFarma® clinical decision support system (Infologic), a platform designed to optimize pharmacological therapy and identify medication‐related risks. NavFarma® cross‐checked drug and diagnostic data and generated alerts for potentially inappropriate medications (PIMs) defined as DDIs, ACB and risks of QTc prolongation and nephrotoxicity. The system also incorporates the 2023 American Geriatrics Society Beers Criteria and the STOPP/START criteria for potentially inappropriate prescription in older people, taking into account patient age, comorbidities and concurrent medications [11]. Clinical pharmacists reviewed the NavFarma® reports and provided structured recommendations including deprescribing, substitution of high‐risk medications, dose adjustment and prioritization of monitoring, within a person‐centred decision‐making process within the EPC (Figure S1).

Physicians were required to actively review these recommendations during the clinical visit, as they were embedded within the visit workflow. Review and acceptance were verified through documentation in the EPC, including explicit notation of accepted recommendations and corresponding therapy modifications (Annex 1).

### Collected variables

In addition to detailed information on medications obtained through the AI system described above, the dataset comprised demographic characteristics and HIV‐related variables such as current and nadir CD4 cell count, CD4/CD8 ratio, time since HIV diagnosis and history of exposure to ART with nucleoside reverse transcriptase inhibitors (NRTIs), non‐nucleoside reverse transcriptase inhibitors (NNRTIs), protease inhibitors (PIs), integrase inhibitors (INSTIs) and boosted regimens. Data on comorbidities and frailty index were also collected. Frailty was assessed using a deficit accumulation frailty index, calculated according to the method proposed by Rockwood and Mitnitski as the ratio of accumulated health deficits to the total number of deficits considered [12]. This study used a 37‐item frailty index (FI) previously validated at MHMC and constructed from health variables collected at the same study visit [13]. Comorbidities were defined according to the latest EACS guidelines and encompassed hypertension, dyslipidaemia, cardiovascular disease, chronic kidney disease, liver disease and osteoporosis [14]. Multimorbidity was defined as the presence of three or more comorbidities.

### Outcomes

The primary outcome was the level of agreement between the medications listed in the EPC and those reported by the patient. Discrepancies were categorized as medications reported by the patient but absent from the EPC or medications listed in the EPC but no longer taken by the patient. Complete agreement was defined as 100% concordance, while values below 100% were considered suboptimal.

Secondary outcomes were the number of actionable alerts generated per patient (PIMs, DDIs, ACB calculated using the Anticholinergic Cognitive Burden (ACB) scale [15], renal toxicity), total pill burden, number of all active pharmacologic agents and HIV‐active agents and the proportion of pharmacist recommendations accepted by physicians and incorporated into patient care plans. An actionable alert for ACB was generated for a cumulative ACB score ≥1.

### Statistical analysis

Continuous variables were summarized as medians (interquartile range, IQR) and categorical variables as counts and percentages. Group comparisons were performed using the Kruskal–Wallis test for continuous variables and the *χ* ^2^ test for categorical variables.

## RESULTS

**FIGURE 1:**
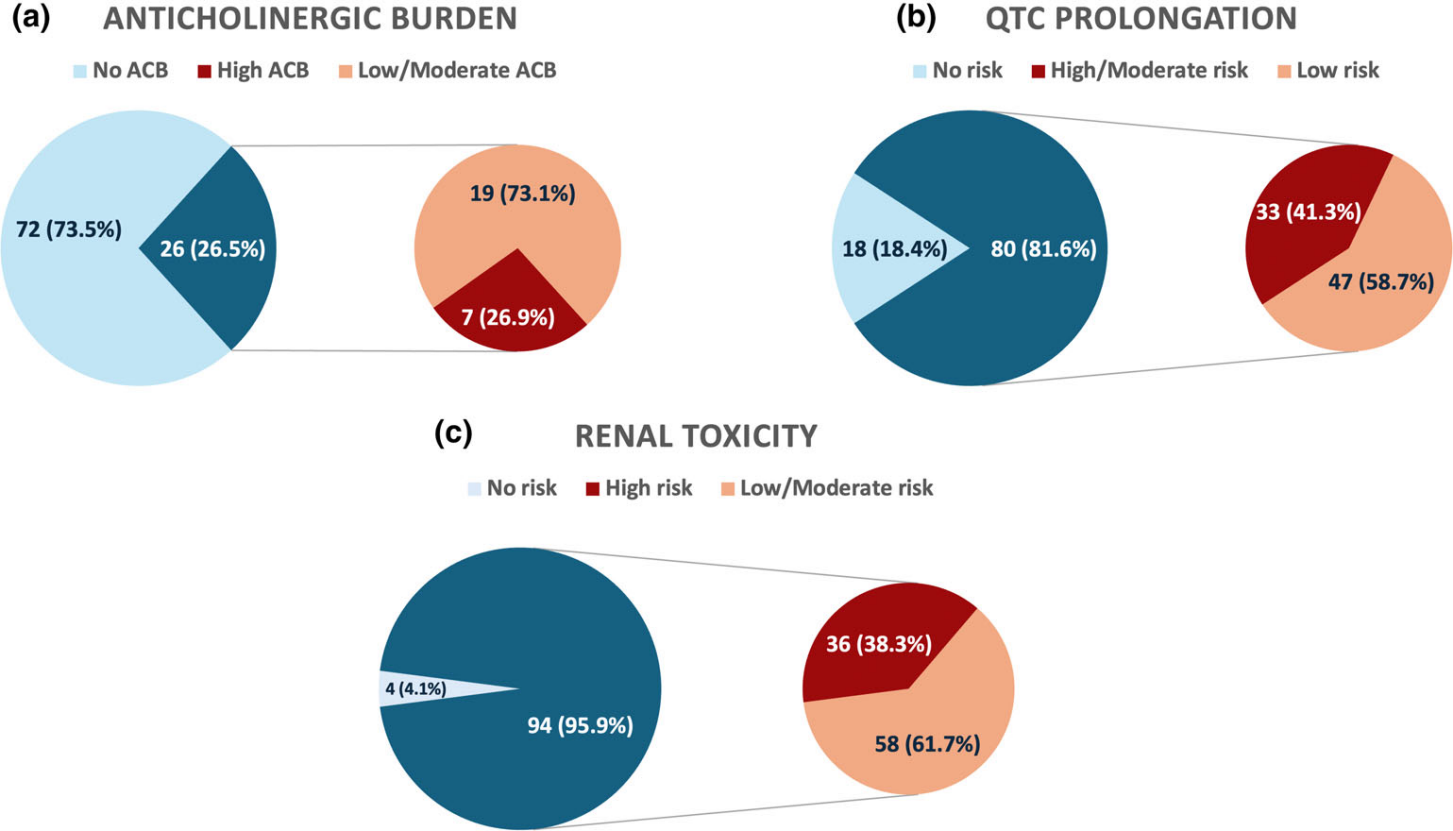
Prevalence of anticholinergic burden (panel a), QTc prolongation risk (panel b) and renal toxicity (panel c).

A total of 181 people with HIV submitted photos of medications from November 2024 to July 2025. Median age was 63 years (IQR, 8), 131 (72%) were male, median nadir CD4 cell count was 200 cells/μL (IQR, 208), current CD4 count was 747 cells/μL (IQR, 395) and median time since HIV diagnosis was 31 years (IQR, 13). In 98 (54.1%) participants, the reconciled medication list was evaluated by a clinical pharmacist for major drug interactions, ACB, and cardiac or renal toxicity. Pharmacist review was targeted to participants with at least one actionable alert identified by the AI‐supported system, reflecting higher pharmacologic complexity. Among these patients, 69 (70.4%) had at least one major drug interaction, 25 (26.5%) had drugs contributing to ACB, 80 (81.6%) had medications associated with QTc prolongation and 94 (95.9%) had drugs linked to nephrotoxicity (Figure 1).

### Primary outcome

Among participants, 111 (61.3%) demonstrated complete agreement between medications documented in the EPC and those reported by the patient, while 70 (38.7%) exhibited discrepancies. Of these, 51 (28.2%) were prescribed medications recorded in the EPC but not reported by patients, while 62 (34.3%) were taking medications not documented in the EPC. Furthermore, 21 (11.6%) initiated new medications, and 19 (10.5%) had medications discontinued during the visit. Overall, the median daily pill burden was 6 (IQR, 4.8), while the total number of concomitant medications (non‐ART drugs) was 10 (IQR, 5).

These undocumented medications most frequently included cardiovascular agents (antihypertensives and low‐intensity lipid‐lowering drugs), proton pump inhibitors, psychotropic agents, supplements and intermittent‐use medications such as non‐steroidal anti‐inflammatory drugs. In most cases, these therapies had been initiated or modified by non‐HIV specialists or primary care providers or were continued by patients beyond the intended duration of treatment. Furthermore, 21 (11.6%) initiated new medications, and 19 (10.5%) had medications discontinued during the visit. Overall, median daily pill burden was 6 (IQR, 4.8), while the total number of concomitant medications (non‐ART drugs) was 10 (IQR, 5).

Pharmacist evaluation identified PIMs in a relevant proportion of participants. The most frequent examples included long‐term proton pump inhibitor use without a clear indication, low‐intensity statins in individuals at high cardiovascular risk, chronic benzodiazepine or sedative‐hypnotic use, anticholinergic agents and chronic non‐steroidal anti‐inflammatory drug use in patients with chronic kidney disease or elevated cardiovascular risk.

Among new prescriptions, 13 (61.9%) were lipid‐lowering agents, 3 (14.3%) were antidepressants and 7 (33.3%) included other drug classes, mainly antihypertensive agents, antidiabetic medications, urological drugs, respiratory agents and vitamin or mineral supplements. Among discontinued drugs, 11 (57.9%) were low‐intensity lipid‐lowering agents, 3 (15.8%) were proton pump inhibitors and 5 (26.3%) included other classes, predominantly antihypertensive agents and supplements. A total of 98 participants were evaluated by the NavFarma® system and a clinical pharmacist. In three (1.7%) cases, medications were discontinued by the pharmacist.

Table 1 presents the comparison between participants with suboptimal agreement (*n* = 70, 38.7%) and those with complete agreement (*n* = 111, 61.3%). No significant differences were observed across demographic or HIV‐related characteristics. Median current and nadir CD4 counts, CD4/CD8 ratio and time since HIV diagnosis were comparable. Exposure to ART classes, including NRTIs, NNRTIs, INSTIs and PIs, was also similar between groups (Table 1).

**TABLE 1:** Characteristics of the study population according to level of agreement between patient‐reported and electronic medication lists.

Variable	Suboptimal agreement, *N* = 70 (38.7%)	Complete agreement, *N* = 111 (61.3%)	*p*
Demographic characteristics			
Age, years, median (Q1, Q3)	62 (59, 66)	64 (60, 67)	0.222
Male sex, *N* (%)	49 (70%)	82 (73.9%)	0.691
HIV‐related variables			
Current CD4 cell count, median (Q1, Q3)	730 (531, 959)	751 (582, 956)	0.686
Nadir CD4 cell count, median (Q1, Q3)	210 (100, 300)	200 (81, 305)	0.925
CD4/CD8 ratio, median (Q1, Q3)	1.42 (0.9, 1.81)	0.85 (0.7, 1.12)	0.078
Time since HIV diagnosis, years, median (Q1, Q3)	32 (25, 36.5)	30.5 (21.75, 36)	0.474
Current exposure to NRTIs, *N* (%)	48 (68.6%)	77 (69.4%)	0.999
Current exposure to NNRTIs, *N* (%)	26 (37.1%)	36 (32.4%)	0.625
Current exposure to INSTIs, *N* (%)	54 (77.1%)	91 (82%)	0.546
Current exposure to PIs, *N* (%)	8 (11.4%)	10 (9%)	0.784
Current exposure to boosters, *N* (%)	6 (8.6%)	10 (9%)	0.999
Comorbidities and frailty			
Hypertension, *N* (%)	36 (51.4%)	47 (42.3%)	0.252
Dyslipidaemia, *N* (%)	55 (78.5%)	93 (83.8%)	0.664
Diabetes mellitus, *N* (%)	4 (5.7%)	13 (11.7%)	0.335
Osteopenia/Osteporosis, *N* (%)	51 (72.9%)	84 (75.7%)	0.757
Multimorbidity, *N* (%)	59 (84.3%)	100 (90.1%)	0.443
Frailty index, median (Q1, Q3)	0.22 (0.18, 0.27)	0.24 (0.19, 0.3)	0.310
Polypharmacy, pill burden and drug interactions			
Daily pill burden, median (Q1, Q3)	7 (4.62, 9)	6 (4, 8.75)	0.180
Number of active agents, median (Q1, Q3)	11 (7, 12)	9 (7, 12)	0.103
Number of HIV‐active agents, median (Q1, Q3)	3 (2, 3)	2 (2, 3)	0.459
Anticholinergic burden score, median (Q1, Q3)	0 (0, 0.25)	0 (0, 1)	0.841
Major interactions, median (Q1, Q3)	2 (1, 4)	1 (0, 3)	0.196
Minor interactions, median (Q1, Q3)	1 (0.75, 2)	1 (0, 2)	0.070
QTc‐prolonging drugs, median (Q1, Q3)	2 (1, 3)	1 (1, 2)	0.052
Potential nephrotoxic drugs, median (Q1, Q3)	2.5 (2, 4)	3 (2, 4)	0.966

With respect to comorbidities and frailty, the prevalence of hypertension, dyslipidaemia, cardiovascular disease, diabetes, chronic kidney disease, liver disease, osteoporosis and multimorbidity did not differ significantly. Frailty index values were also comparable between groups. No significant differences were detected in pill burden, number of concomitant medications (non‐ART drugs) or drug interaction measures (Table 1).

### Pill burden

Supplementary Table S1 shows that participants with higher pill burden (≥6 pills/day) had longer HIV duration, were more frequently exposed to protease inhibitors and boosters and had a higher prevalence of diabetes compared with those with lower pill burden. The frailty index was significantly higher in the high pill burden group, which also had greater numbers of concomitant medications, more HIV‐active agents, higher ACB and more frequent major and minor interactions, as well as higher exposure to QTc‐prolonging and nephrotoxic drugs (Table S1).

### Active pharmacologic agents

Table S2 presents the study population according to the number of total active pharmacologic agents. Participants with ≥10 prescribed drugs more often had hypertension and a higher frailty index compared with those with fewer drugs. They also had a greater daily pill burden, more HIV‐active agents and lower agreement with the treating physician. Polypharmacy‐related risks, including major and minor interactions, QTc‐prolonging drugs and nephrotoxic drugs, were also higher in this group (Table S2).

### DDIs and pharmacologic risks

**FIGURE 2:**
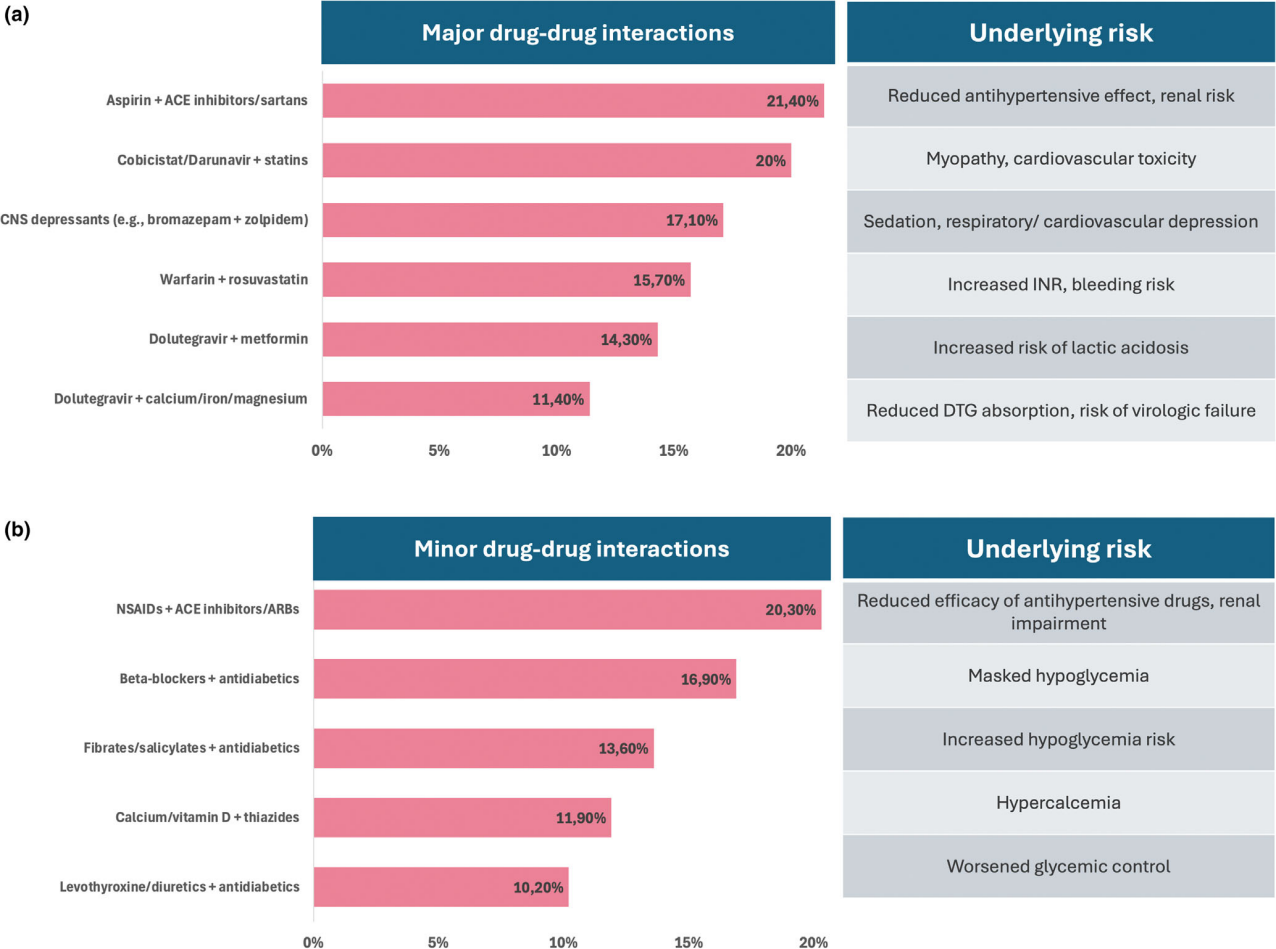
Major (panel a) and minor (panel b) drug–drug interactions (DDIs) identified through pharmacist and AI‐supported review. Major interactions were defined as those with potential for serious clinical consequences requiring therapy modification; minor interactions were defined as those of lower clinical impact but requiring monitoring.

Following integration of AI‐supported medication analysis and pharmacist review, 70 clinically relevant major DDIs and 59 minor interactions were identified. Major DDIs frequently involved central nervous system depressants, pharmacokinetic interactions with antiretrovirals (notably dolutegravir and cobicistat‐boosted regimens), cardiovascular agents (ACE inhibitors, sartans, aspirin), anticoagulants and statins (Figure 2a). Minor DDIs most often affected glycaemic control, blood pressure regulation and calcium metabolism (Figure 2b).

**FIGURE 3:**
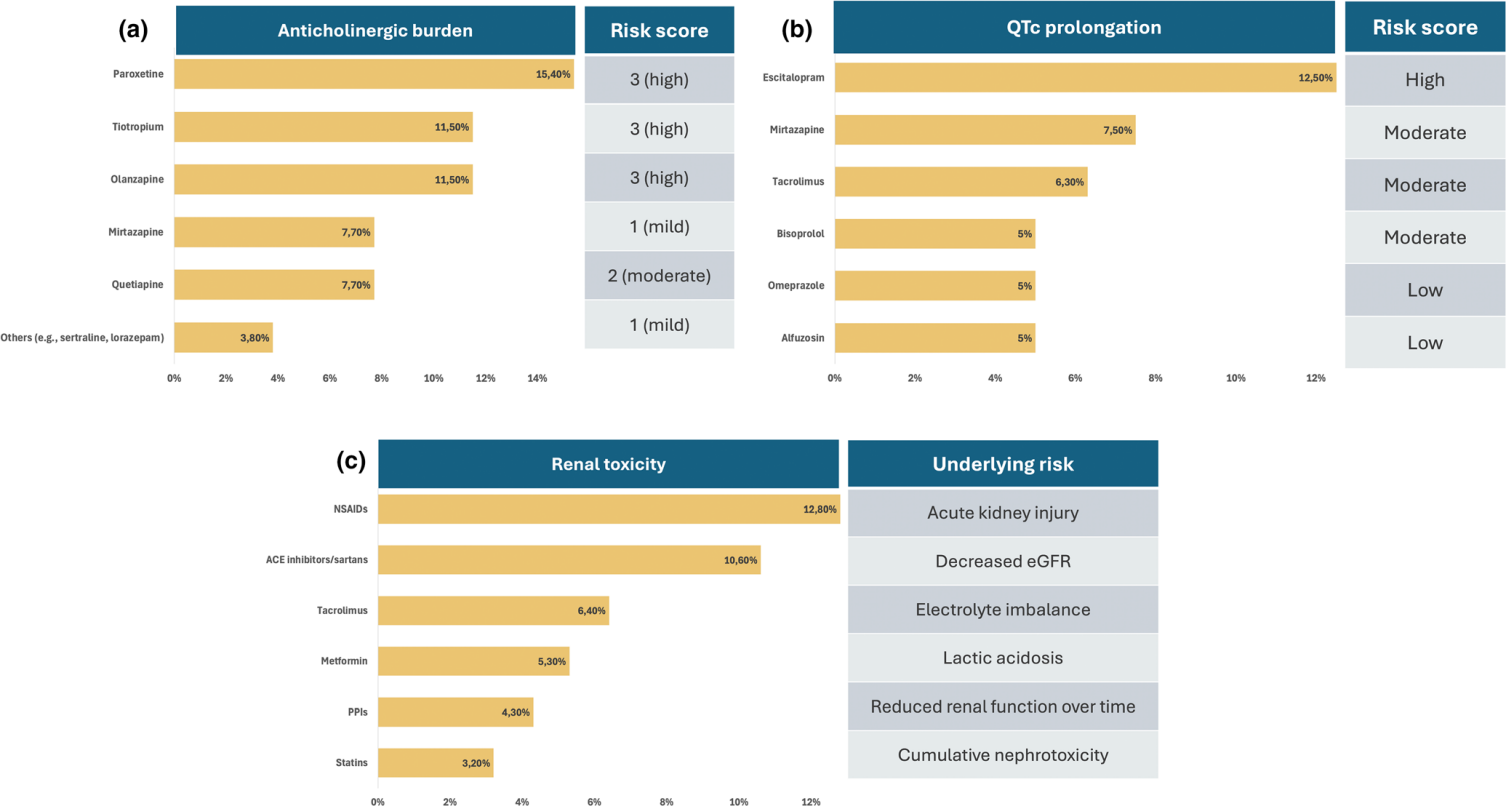
Drugs associated with increased anticholinergic burden (panel a), QTc prolongation risk (panel b) and renal toxicity (panel c). Listed drugs represent those most frequently contributing to cumulative pharmacologic risks identified through the AI‐supported NavFarma® system and pharmacist review.

Regarding ACB, 26.9% of reviewed cases showed high cumulative risk (Figure 1a), mainly due to psychotropic or respiratory agents (Figure 3a). QTc prolongation risk classified as high or moderate was identified in 41.3% of cases (Figure 1c), with common agents including escitalopram, mirtazapine, tacrolimus and alfuzosin (Figure 3b). In our study, renal toxicity was flagged in 38.3% of cases (Figure 1c). Renal toxicity alerts reflected the presence of potentially nephrotoxic medications in patients with impaired or borderline renal function, based on routinely monitored serum creatinine and estimated glomerular filtration rate, rather than observed acute renal events, most frequently linked to NSAIDs, ACE inhibitors/sartans, tacrolimus, metformin, proton pump inhibitors and statins (Figure 3c).

All pharmacist observations were recorded in the EPC and linked to actionable suggestions, which were subsequently reviewed by clinicians to guide therapy optimization in aging people with HIV.

## DISCUSSION

This QI intervention at MHMC demonstrated both feasibility and acceptability within a real‐world clinical setting of older people with HIV. The photo‐based submission approach enabled precise reconciliation of drug lists by identifying undocumented and discontinued medications, creating opportunities for therapeutic optimization. Nearly 40% of participants had discrepancies between EPC records and actual drug use, a prevalence consistent with prior studies in aging people with HIV that emphasize the frequency of undocumented medications and their clinical impact. Importantly, these mismatches were not explained by age, sex, immunologic status or time since HIV diagnosis, likely reflecting the complexity of polypharmacy and the fragmentation of care when multiple specialists are involved [16].

Similar challenges have been reported in other cohorts of aging people with HIV across Europe, where undocumented drugs or supplements increased the risk of clinically significant DDIs [17]. For example, in a French cross‐sectional study of 496 people with HIV, 32% were found to have unknown co‐medications [17], while in the Italian gestione ambulatoriale politerapie cohort of 556 people with HIV, undocumented treatments were linked to increased DDI risk, virologic failure and drug toxicities [18]. These results reinforce the global challenge of polypharmacy, highlighting the need for harmonized strategies promoted by the WHO Medication Without Harm programme [19] and EACS guidelines [14].

Most discrepancies in our study involved within‐class substitutions or dose modifications, suggesting that systematic reconciliation, rather than single interventions, can ensure longitudinal safety. The most common new prescriptions were high‐ or moderate‐intensity lipid‐lowering agents, while low‐intensity statins were most frequently discontinued, reflecting adaptation of prescribing to evolving evidence and cardiovascular prevention algorithms [20]. These findings resonate with recent updates in HIV and cardiovascular guidelines following the REPRIEVE trial, which demonstrated statin efficacy in people with HIV with low‐to‐moderate cardiovascular risk [21].

The novelty of this QI project lies in integrating AI‐driven drug recognition into the EPC, combined with patient engagement via familiar digital tools such as WhatsApp. This not only increased accuracy but also promoted active patient participation, consistent with literature advocating participatory approaches to drug reconciliation in older populations [22, 23, 24]. This low‐barrier, scalable model could be implemented across diverse healthcare systems to strengthen digital health integration and reduce prescribing errors globally. Engaging patients helps capture nonadherence, discontinued therapies and over‐the‐counter medications, factors often overlooked in standard prescribing but crucial to safety in people with HIV [17, 18].

Despite reconciliation efforts and reduced use of boosted regimens, DDIs remain relevant in people with HIV, with 3% classified as red‐flag interactions [25]. In our analysis, the definition of DDIs encompassed both major and minor interactions based on pharmacological criteria. This means that not all identified interactions are necessarily harmful or require therapeutic discontinuation, but rather may indicate the need for closer monitoring or potential dose adjustments. In this scenario, polypharmacy in older people with HIV with DDIs is often unavoidable due to multimorbidity and may be either appropriate or inappropriate depending on comprehensive medication review and individualized risk–benefit assessment. A typical clinical example, as also confirmed in our study, is the interaction between statins and boosted protease inhibitors in an older patient with a long history of HIV, prior virologic failures and elevated cardiovascular risk [26]. This interaction creates a therapeutic dilemma, as the ability to intensify statin therapy to achieve optimal low‐density lipoprotein (LDL) cholesterol reduction is constrained by safety concerns and may further exacerbate statin hesitancy, which already represents a challenge in clinical practice [27, 28, 29].

On the other hand, certain DDIs or inappropriate medications may go unrecognized by physicians and increase the risk of drug toxicities, including QTc prolongation, ACB and nephrotoxicity, all of which were evaluated in our study. QTc prolongation is a relevant concern. In the present system, QTc risk reflects cumulative exposure to QTc‐prolonging drugs rather than formal modelling of additive or synergistic pharmacodynamic effects, which represents an important future development. In our study, 81.6% of people with HIV were exposed to drugs with the potential to prolong the QTc interval, of which 41.3% had moderate‐to‐high risk of QTc prolongation. However, we were unable to perform ECGs on all participants to determine the actual prevalence of QTc prolongation. Previous studies have reported prevalence rates between 10% and 23% in people with HIV, depending on the definition applied and the demographic characteristics of the cohort [30]. Moreover, ACB represents a significant issue in people with HIV, as it contributes to the cumulative risk of frailty, falls and cognitive decline [31]. In our cohort, 26% of patients had an ACB score greater than 0, with the most frequently implicated drugs being central nervous system agents used to treat transitory depressive episodes that do not need long‐term treatment. Furthermore, chronic kidney disease increases with advancing age in the general population, and people with HIV may additionally be exposed to ART with nephrotoxic potential. When combined with other medications such as NSAIDs, ACE inhibitors and sartans, this further increases the risk of chronic kidney disease. In our study, renal toxicity was flagged in 38.3% of cases. The frequency of renal toxicity exacerbated by DDIs in people with HIV is not precisely quantified in the medical literature.

Taken together, our results emphasize the importance of systematic medication reconciliation and structured pharmacist input in aging people with HIV. Pharmacists' involvement provided an additional safeguard, identifying inappropriate medications and suggesting deprescribing strategies aligned with geriatric care principles. Deprescribing and simplification are crucial for reducing cumulative toxicity. HIV‐specific studies have demonstrated that polypharmacy and inappropriate prescribing in people with HIV are associated with increased risks of DDIs, hospitalizations and healthcare utilization, underscoring the need for targeted medication optimization strategies in this population [32, 33].

However, several limitations of this study should be acknowledged. First, the project is currently single‐centre and cross‐sectional, which limits the generalizability of the findings. Second, data collection was not longitudinal, and clinical outcomes, such as reductions in drug toxicities or improvements in adherence, have not yet been evaluated. Third, the study population consisted of older people with HIV who had a high burden of multimorbidity and polypharmacy, which may limit the applicability of the findings to the broader people with HIV population. Fourth, the study may not fully capture the complexity of prescribing practices in older people with HIV without access to the internet or other digital tools, meaning that polypharmacy in the most vulnerable individuals could not be adequately assessed. Key strengths of the model presented in this study include the automation of medication input, seamless integration with the electronic health record and the emphasis on multidisciplinary care. By automating medication collection and reconciliation, the intervention reduces manual workload and concentrates human involvement on higher‐value activities, suggesting potential healthcare delivery efficiencies and downstream cost savings. The average pharmacist time required to review each AI‐generated report and produce clinical recommendations was approximately 50–60 min per selected high‐risk patient. Although this represents a considerable time investment, the AI‐generated reports substantially facilitated the review process and reduced the time that would have been required to generate such comprehensive assessments manually. At the same time, pharmacist oversight remained essential to critically review, interpret and contextualize the AI‐generated outputs within the clinical setting.

These findings suggest that combining AI‐supported medication reconciliation with pharmacist‐led review improves polypharmacy management in aging people with HIV. This approach enhances medication accuracy, supports the identification of potentially inappropriate prescribing and clinically relevant DDIs and aligns HIV care with person‐centred, multidisciplinary strategies informed by emerging evidence from both HIV and geriatric medicine. Building on these results, future work will pursue complementary directions: further engineering the tool as a scalable instrument for clinical implementation; using the generated evidence to inform hospital leadership about the structural need for dedicated clinical pharmacists within HIV outpatient settings; increasingly leveraging AI tools natively integrated into the EPC; and evaluating the long‐term impact of pharmacist‐led interventions supported by AI. Importantly, this study underscores that the core value of the intervention lies not in AI alone, but in the strengthened and sustained collaboration with the clinical pharmacist, with AI acting as an enabler of more effective multidisciplinary dialogue and a scalable, safer model of HIV care.

## AUTHOR CONTRIBUTIONS

JM, AP and GG conceptualized and designed the manuscript. JM, CR and GG wrote and revised the manuscript. PDG generated and AP reviewed all NavPharma reports. GLL and CG enabled AI‐enhanced medication data collection and automated drug reconciliation. JM did the statistical analysis. JM, AP, ChiM, CriM, CG and GG did the supervision of the final version of the manuscript. All the authors contributed to discussion and revised the manuscript.

## FUNDING INFORMATION

This study was supported by an unconditional grant provided by Merck Italy.

## CONFLICT OF INTEREST STATEMENT

JM received speaker honoraria from Gilead and ViiV. GG and CriM received research grant and speaker honorarium from Gilead, ViiV, MERCK and Jansen. GG and CM attended advisory boards of Gilead, ViiV and MERCK. Other authors reported no conflict of interest.

## Supporting information


**Figure S1.** Steps of the quality improvement (QI) project.
**Table S1**. Characteristics of the study population according to pill burden. Participants were divided into two groups: lower pill burden (<6 daily pills) and higher pill burden (≥6 daily pills). Data are expressed as median (Q1–Q3) or number (%).
**Table S2**. Characteristics of the study population according to the number of total prescribed drugs. Participants were classified as having a lower number of prescribed drugs (<10) or a higher number (≥10). Data are expressed as median (Q1–Q3) or number (%).

## Data Availability

The data that support the findings of this study are available on request from the corresponding author. The data are not publicly available due to privacy or ethical restrictions.

## References

[1] Palella FJ , Hart R , Armon C , et al. Non‐AIDS comorbidity burden differs by sex, race, and insurance type in aging adults in HIV care. Aids. 2019;33:2327‐2335. 31764098 10.1097/QAD.0000000000002349 PMC12329769

[2] Guaraldi G , Malagoli A , Calcagno A , et al. The increasing burden and complexity of multi‐morbidity and polypharmacy in geriatric HIV patients: a cross sectional. BMC Geriatr. 2018;18:1‐10. 29678160 10.1186/s12877-018-0789-0 PMC5910563

[3] Marcus JL , Leyden WA , Alexeeff SE , et al. Comparison of overall and comorbidity‐free life expectancy between insured adults with and without HIV infection, 2000‐2016. JAMA Netw Open. 2020;3:e207954. 32539152 10.1001/jamanetworkopen.2020.7954 PMC7296391

[4] Guaraldi G , Orlando G , Zona S , et al. Premature age‐related comorbidities among HIV‐infected persons compared with the general population. Clin Infect Dis. 2011;53:1120‐1126. 21998278 10.1093/cid/cir627

[5] Deutschmann E , Bucher HC , Jaeckel S , et al. Prevalence of potential drug‐drug interactions in patients of the swiss HIV cohort study in the era of HIV integrase inhibitors. Clin Infect Dis. 2021;73:E2145‐E2152. 32634832 10.1093/cid/ciaa918

[6] 2023 American Geriatrics Society Beers Criteria® Update Expert Panel . American Geriatrics Society 2023 updated AGS beers criteria® for potentially inappropriate medication use in older adults. J Am Geriatr Soc. 2023;71:2052‐2081. 37139824 10.1111/jgs.18372 PMC12478568

[7] Sukumaran L , Winston A , Marzolini C , et al. Polypharmacy in HIV: rethinking what counts and why it matters. HIV Med. 2025;27:186‐199. 41121455 10.1111/hiv.70129 PMC12861129

[8] Orenstein L , Chetrit A , Laufer K , Dankner R . A prospective study on potentially inappropriate drug use and all‐cause mortality in community‐dwelling older adults. J Am Geriatr Soc. 2025;73:2828‐2838. 40785370 10.1111/jgs.70002 PMC12460961

[9] Alaa Eddine N , Schreiber J , El‐Yazbi AF , Shmaytilli H , Amin MEK . A pharmacist‐led medication review service with a deprescribing focus guided by implementation science. Front Pharmacol. 2023;14:1097238. 36794277 10.3389/fphar.2023.1097238 PMC9922726

[10] Gray SL , Perera S , Soverns T , Hanlon JT . Systematic review and meta‐analysis of interventions to reduce adverse drug reactions in older adults: an update. Drugs Aging. 2023;40:965‐979. 37702981 10.1007/s40266-023-01064-y PMC10600043

[11] American Geriatrics Society . Updated AGS beers criteria® for potentially inappropriate medication use in older adults. J Am Geriatr Soc. 2023;2023(71):2052‐2081. 10.1111/jgs.18372 PMC12478568 37139824

[12] Rockwood K , Mitnitski A . Frailty in relation to the accumulation of deficits. J Gerontol A Biol Sci Med Sci. 2007;62:722‐727. 17634318 10.1093/gerona/62.7.722

[13] Guaraldi G , Brothers TD , Zona S , et al. A frailty index predicts survival and incident multimorbidity independent of markers of HIV disease severity. Aids. 2015;29:1633‐1641. 26372273 10.1097/QAD.0000000000000753

[14] Ambrosioni J , Levi L , Alagaratnam J , et al. Major revision version 12.0 of the European AIDS clinical society guidelines 2023. HIV Med. 2023;24:1126‐1136. 37849432 10.1111/hiv.13542

[15] Boustani M , Campbell N , Munger S , Maidment I , Fox C . Impact of anticholinergics on the aging brain: a review and practical application. Aging Health. 2008;4:311‐320.

[16] Prior A , Vestergaard CH , Vedsted P , et al. Healthcare fragmentation, multimorbidity, potentially inappropriate medication, and mortality: a Danish nationwide cohort study. BMC Med. 2023;21:305. 37580711 10.1186/s12916-023-03021-3 PMC10426166

[17] Tetart M , Passecountrin P , Lesourd A , et al. Are unknown co‐medications, over‐the‐counter and off‐label drug use still problems among people living with HIV? Results from a transversal survey in 23 centres in France. J Antimicrob Chemother. 2023;78:2731‐2734. 37757452 10.1093/jac/dkad292

[18] Cattaneo D , Oreni L , Meraviglia P , et al. Polypharmacy and aging in people living with HIV: 6 years of experience in a multidisciplinary outpatient clinic. Drugs Aging. 2023;40:665‐674. 37310576 10.1007/s40266-023-01037-1

[19] WHO Medication Without Harm . 2024. https://www.who.int/initiatives/medication-without-harm

[20] Alagaratnam J , van Bremen K , Behrens GMN , et al. Statin use in HIV: European AIDS clinical society guidance for the primary prevention of cardiovascular disease. Lancet HIV. 2025;12:e382‐e392. 40316403 10.1016/S2352-3018(25)00047-5

[21] Grinspoon KS , Fitch VK , Zanni VM , et al. Pitavastatin to prevent cardiovascular disease in HIV infection. N Engl J Med. 2023;389:687‐699. doi:10.1056/NEJMoa2304146 37486775 PMC10564556

[22] Doucette L , Kiely BT , Gierisch JM , et al. Participatory research to improve medication reconciliation for older adults in the community. J Am Geriatr Soc. 2023;71:620‐631. 36420635 10.1111/jgs.18132 PMC9957786

[23] Prey JE , Polubriaginof F , Grossman LV , et al. Engaging hospital patients in the medication reconciliation process using tablet computers. J Am Med Inform Assoc. 2018;25:1460‐1469. 30189000 10.1093/jamia/ocy115 PMC7263785

[24] Heyworth L , Paquin AM , Clark J , et al. Engaging patients in medication reconciliation via a patient portal following hospital discharge. J Am Med Inform Assoc. 2013;21:e157. 24036155 10.1136/amiajnl-2013-001995 PMC3957401

[25] López‐Centeno B , Badenes‐Olmedo C , Mataix‐Sanjuan Á , et al. Polypharmacy and drug‐drug interactions in people living with human immunodeficiency virus in the region of Madrid, Spain: a population‐based study. Clin Infect Dis. 2020;71:353‐362. 31428770 10.1093/cid/ciz811

[26] Mazzitelli M , Pontillo D , Clemente T , et al. Polypharmacy, anticholinergic burden and drug‐drug interaction assessment in people with four‐class‐resistant HIV: data from the PRESTIGIO registry. J Antimicrob Chemother. 2024;79:2163‐2169. 39001781 10.1093/jac/dkae190

[27] Brown CJ , Chang LS , Hosomura N , et al. Assessment of sex disparities in nonacceptance of statin therapy and low‐density lipoprotein cholesterol levels among patients at high cardiovascular risk. JAMA Netw Open. 2023;6:e231047. 36853604 10.1001/jamanetworkopen.2023.1047 PMC9975905

[28] Gallegos Aragon K , Ray G , Conklin J , et al. Underprescribing of statin therapy in people with HIV at risk for atherosclerotic cardiovascular disease. Am J Health‐Syst Pharm. 2022;79:2026‐2031. 35976174 10.1093/ajhp/zxac224

[29] Ladapo JA , Richards AK , DeWitt CM , et al. Disparities in the quality of cardiovascular care between HIV‐infected versus HIV‐uninfected adults in the United States: a cross‐sectional study. J Am Heart Assoc. 2017;6:e007107. 29138182 10.1161/JAHA.117.007107 PMC5721786

[30] Chastain DB , Pveve M , Wagner JL . Abnormal QTc syndrome in HIV‐infected patients: a systematic review of prevalence and risk factors. Antivir Ther. 2019;24:459‐465. 31570667 10.3851/IMP3335

[31] Mazzitelli M , Trunfio M , Coin A , et al. Use of different anticholinergic scales and their correlation with anticholinergic symptom burden in a cohort of people living with HIV. J Antimicrob Chemother. 2024;79:66‐77. 37965917 10.1093/jac/dkad348 PMC11032244

[32] Guaraldi G , Milic J , Marcotullio S , Mussini C . A patient‐centred approach to deprescribing antiretroviral therapy in people living with HIV. J Antimicrob Chemother. 2020;75:3425‐3432. 32747939 10.1093/jac/dkaa329

[33] Blanco J , Morillo R , Abril V , et al. Deprescribing of Non‐antiretroviral Therapy in HIV‐Infected Patients. Eur J Clin Pharmacol. 2020;76:305‐318. 31865412 10.1007/s00228-019-02785-z

