# Supplementary material for: A multidisciplinary, AI‐supported quality improvement intervention to manage polypharmacy in aging people with HIV
Source: HIV Med. 2026 Apr 11;27(7):1109–19. doi: 10.1111/hiv.70234 (PMC13340986; doi:10.1111/hiv.70234)
Supplement: Supplementary file 1 — Figure S1. Steps of the quality improvement (QI) project. Table S1. Characteristics of the study population according to pill burden. Participants were divided into two groups: lower pill burden (<6 daily pills) and higher pill burden (≥6 daily pills). Data are expressed as median (Q1–Q3) or number (%). Table S2. Characteristics of the study population according to the number of total prescribed drugs. Participants were classified as having a lower number of prescribed drugs (<10) or a higher number (≥10). Data are expressed as median (Q1–Q3) or number (%). [file HIV-27-1109-s001.docx]

**Annex 1. Overview of the Quality Improvement Project**

***(I) AI Drug Recognition and Reconciliation.*** Patients received an automated message one week before their visit, inviting them to upload pictures of their medications via WhatsApp. These images, focused on box or barcode labels, were processed by AI systems embedded in the electronic patient chart (EPC), which recognized and translated them into Anatomical Therapeutic Chemical (ATC) codes. Patients confirmed the dosage through a guided interface. During the clinical visit, the physician validated the medication list, indicating whether each drug was previously prescribed (and, if so, the starting date) or is new.

***(II) AI Integration with NavFarma Database.*** NavFarma® is a clinical decision support platform developed by Infologic, designed to assist healthcare providers in optimizing pharmacological therapy and preventing medication-related risks. The system integrates multiple data sources, including outpatient prescriptions, hospital discharges, and direct drug distribution and applies a continuously updated knowledge base. This includes tools such as the Micromedex® Drug Interactions database, Beers START and STOPP criteria, and evaluations of anticholinergic burden, nephrotoxicity, and QT prolongation risks.

In this study, NavFarma® received standardized medication data (ATC codes) and patient comorbidities (ICD-10 codes) directly from the MHMC electronic record. It generated a structured electronic report for each patient highlighting: potentially inappropriate medications, major and minor drug-drug Interactions (DDIs), anticholinergic cognitive burden (ACB) score, kidney and cardiac toxicity alerts, including QT prolongation, adherence issues and deprescription opportunities. This report was then reviewed by clinical pharmacists, whose recommendations were integrated into the medical record to guide clinicians in therapy optimization.

***(III) Pharmacist review and recommendations.*** Clinical pharmacists reviewed the NavFarma® reports and translated them into structured recommendations within the EPC. These suggestions included proposals to discontinue, adjust, or replace medications, with clinical rationale provided for each.

**(IV)** ***Multidisciplinary decision-making.*** During the clinical visit, physicians reviewed pharmacist recommendations and determined whether to adopt them based on clinical judgment and patient-specific considerations.

**Annex 2: AI-Supported Medication Collection Tool**

***Overview***

This supplementary document describes the prototype software used to collect and process medication data for the AI-supported quality improvement project at the Modena HIV Metabolic Clinic (MHMC). The system, named “ Medication record system” was designed to allow PWH to report their current medications easily via **WhatsApp**, avoiding the need for a dedicated app or web portal. WhatsApp was selected due to its widespread use in Italy (37.5 million users, ~70% of the population over 10 years old, ISTAT 2024).

***User Interaction***

Patients received written instructions via WhatsApp inviting them to photograph all medications currently taken, including prescription drugs, over-the-counter medications, supplements, and therapies prescribed by other healthcare providers. Patients were instructed to send one photo per medication (preferably of the box or label) and to specify the daily dosage or administration schedule using a short text message. Upon receiving a photo, the system confirms receipt and requests dosage information (e.g., “one tablet per day,” “six drops a day,” “one ampoule at the beginning of each month,” “half a tablet per day”).

***Drug Recognition***

The software processes the submitted images using **optical character recognition (OCR)** based on neural networks. Since pharmacy barcodes are often removed, text extraction replaces barcode scanning. The recognized text is tokenized and matched to a national database of more than 20,000 commercial drug names. A scoring algorithm ranks possible matches.

***Technical Implementation***

The prototype was built using:

- **WhatsApp Business API** for user interaction
- **Python/Django** for server logic and web interface
- **RabbitMQ** and **Celery** for message queue and asynchronous task management
- **SQLite** for local database storage
- **JSON-based REST API** for integration with the MHMC electronic patient chart

Example JSON output for one patient entry:

{

"wa_id": "39123456789",

"name": "Sample User",

"time_sent": "2024-10-04T19:44:08Z",

"image": "/path/to/image/userid/984675394.jpg",

"code": "044628016",

"dosage": "one tablet per day"

}

The tool successfully enabled medication data collection and AI-based recognition through an accessible, low-barrier communication channel. Its integration with the MHMC clinical system allowed real-time, structured medication reconciliation supporting the quality improvement intervention.

***Supplementary figure 1.*** *Steps of the quality improvement (QI) project.*

***
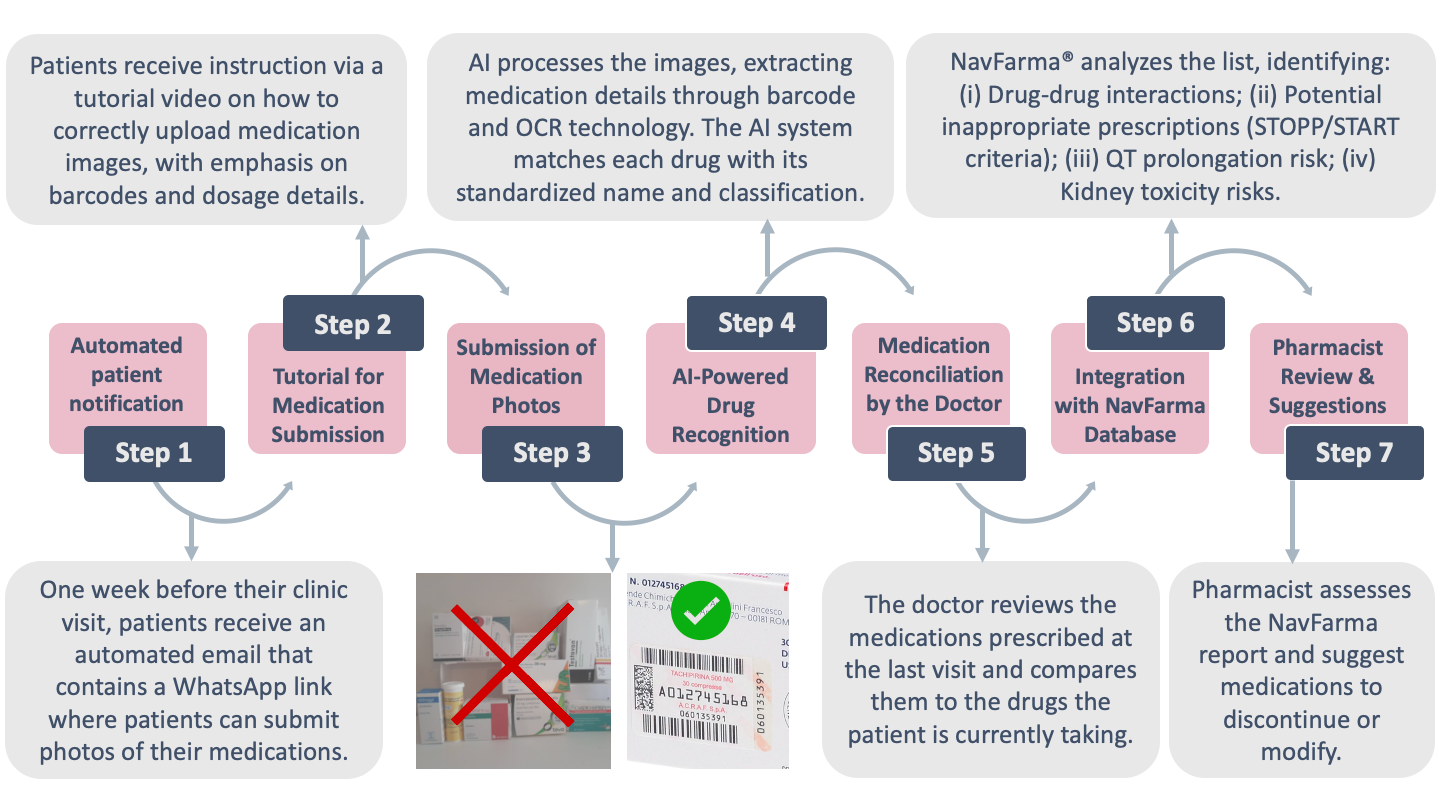
***

***Supplementary table 1*. Characteristics of the study population according to pill burden.** Participants were divided into two groups: lower pill burden (<6 daily pills) and higher pill burden (≥6 daily pills). Data are expressed as median (Q1–Q3) or number (%).

| **Variable** | **Lower pill burden**  **N = 71 (39.2%)** | **Higher pill burden**  **N = 110 (60.8%)** | **p** |
| --- | --- | --- | --- |
| **Demographic characteristics** | | | |
| Age, years, median (Q1, Q3) | 62 (58, 66) | 63 (59.25, 67.75) | 0.181 |
| Male sex, N (%) | 54 (76.1%) | 77 (70%) | 0.472 |
| **HIV-related variables** | | | |
| Current CD4 cell count, median (Q1, Q3) | 777.5 (579.75, 973.25) | 704.5 (562, 949) | 0.550 |
| Nadir CD4 cell count, median (Q1, Q3) | 202 (100, 310) | 200 (89, 299) | 0.645 |
| CD4/CD8 ratio, median (Q1, Q3) | 0.71 (0.7, 1.16) | 1.01 (0.79, 1.45) | 0.305 |
| Time since HIV diagnosis, years, median (Q1, Q3) | 27 (20, 35) | 32 (26, 37) | **<0.001** |
| Current exposure to NRTIs, N (%) | 54 (76.1%) | 71 (64.5%) | 0.141 |
| Current exposure to NNRTIs, N (%) | 19 (26.8%) | 43 (39.1%) | 0.122 |
| Current exposure to INSTIs, N (%) | 60 (84.5%) | 85 (77.3%) | 0.317 |
| Current exposure to PIs, N (%) | 2 (2.8%) | 16 (14.5%) | **0.020** |
| Current exposure to boosters, N (%) | 1 (1.4%) | 15 (13.6%) | **0.010** |
| **Comorbidities and frailty** | | | |
| Hypertension, N (%) | 28 (39.4%) | 55 (50%) | 0.363 |
| Dyslipidemia, N (%) | 56 (78.9%) | 92 (83.6%) | 0.707 |
| Diabetes mellitus, N (%) | 1 (1.4%) | 16 (14.5%) | **0.012** |
| Osteopenia/Osteoporosis, N (%) | 51 (71.8%) | 84 (76.4%) | 0.736 |
| Multimorbidity, N (%) | 60 (84.5%) | 99 (90%) | 0.475 |
| Frailty index, median (Q1, Q3) | 0.22 (0.14, 0.24) | 0.27 (0.19, 0.3) | **<0.001** |
| **Polypharmacy, pill burden and drug interactions** | | | |
| Agreement, median (Q1, Q3) | 100 (70.5, 100) | 100 (57.75, 100) | 0.158 |
| Number of active agents, median (Q1, Q3) | 7 (6, 8) | 11.5 (10, 13.75) | **<0.001** |
| Number of HIV active agents, median (Q1, Q3) | 2 (2, 3) | 3 (2, 3) | **0.038** |
| Anticholinergic burden score, median (Q1, Q3) | 0 (0, 0) | 0 (0, 1) | **<0.001** |
| Major interactions, median (Q1, Q3) | 0 (0, 1) | 3 (1, 4) | **<0.001** |
| Minor interactions, median (Q1, Q3) | 0 (0, 1) | 1 (1, 2) | **<0.001** |
| QTc‑prolonging drugs, median (Q1, Q3) | 1 (0, 1) | 2 (1, 3) | **<0.001** |
| Potential nephrotoxic drugs, median (Q1, Q3) | 2 (1, 3) | 3 (2, 4) | **0.007** |

***Supplementary table 2*. Characteristics of the study population according to the number of total prescribed drugs.** Participants were classified as having a lower number of prescribed drugs (<10) or a higher number (≥10). Data are expressed as median (Q1–Q3) or number (%)

| **Variable** | **Lower number of AA**  **N = 89 (49.2%)** | **Higher number of AA**  **N = 92 (50.8%)** | **p** |
| --- | --- | --- | --- |
| **Demographic characteristics** | | | |
| Age, years, median (Q1, Q3) | 62 (59, 66) | 64 (59, 68) | 0.159 |
| Male sex, N (%) | 66 (74.2%) | 65 (70.7%) | 0.718 |
| **HIV-related variables** | | | |
| Current CD4 cell count, median (Q1, Q3) | 754 (601, 970.25) | 712 (538.5, 950.25) | 0.551 |
| Nadir CD4 cell count, median (Q1, Q3) | 216 (109.5, 300) | 180 (76, 300) | 0.405 |
| CD4/CD8 ratio, median (Q1, Q3) | 0.91 (0.69, 1.12) | 0.9 (0.74, 1.42) | 0.269 |
| Time since HIV diagnosis, years, median (Q1, Q3) | 31 (21, 36) | 32 (25, 36) | 0.424 |
| Current exposure to NRTIs, N (%) | 60 (67.4%) | 65 (70.7%) | 0.757 |
| Current exposure to NNRTIs, N (%) | 29 (32.6%) | 33 (35.9%) | 0.757 |
| Current exposure to INSTIs, N (%) | 74 (83.1%) | 71 (77.2%) | 0.412 |
| Current exposure to PIs, N (%) | 7 (7.9%) | 11 (12%) | 0.502 |
| Current exposure to boosters, N (%) | 6 (6.7%) | 10 (10.9%) | 0.474 |
| **Comorbidities and frailty** | | | |
| Hypertension, N (%) | 33 (37.1%) | 50 (54.3%) | **0.023** |
| Dyslipidemia, N (%) | 74 (83.1%) | 74 (80.4%) | 0.787 |
| Diabetes mellitus, N (%) | 4 (4.5%) | 13 (12.1%) | 0.055 |
| Osteopenia/Osteoporosis, N (%) | 70 (78.7%) | 65 (70.7%) | 0.466 |
| Multimorbidity, N (%) | 79 (88.8%) | 80 (87%) | 0.710 |
| Frailty index, median (Q1, Q3) | 0.22 (0.16, 0.24) | 0.27 (0.22, 0.32) | **<0.001** |
| **Polypharmacy, pill burden and drug interactions** | | | |
| Agreement, median (Q1, Q3) | 100 (80, 100) | 100 (55.25, 100) | **0.023** |
| Daily pill burden, median (Q1, Q3) | 4.25 (4, 6) | 9 (7, 11) | **<0.001** |
| Number of HIV active agents, median (Q1, Q3) | 2 (2, 3) | 3 (2, 3) | **0.014** |
| Anticholinergic burden score, median (Q1, Q3) | 0 (0, 0) | 0 (0, 1) | 0.101 |
| Major interactions, median (Q1, Q3) | 1 (0, 1) | 3 (1, 4) | **<0.001** |
| Minor interactions, median (Q1, Q3) | 0 (0, 1) | 1 (1, 2.25) | **<0.001** |
| QTc‑prolonging drugs, median (Q1, Q3) | 1 (0, 2) | 2 (1, 3) | **<0.001** |
| Potential nephrotoxic drugs, median (Q1, Q3) | 2 (1, 3) | 3 (2, 4) | **<0.001** |
